# iNOS Interacts with Autophagy Receptor p62 and is Degraded by Autophagy in Macrophages

**DOI:** 10.3390/cells8101255

**Published:** 2019-10-15

**Authors:** Jing Wang, Ming-Yue Wu, Huanxing Su, Jinjian Lu, Xiuping Chen, Jieqiong Tan, Jia-Hong Lu

**Affiliations:** 1 State Key Laboratory of Quality Research in Chinese Medicine, Institute of Chinese Medical Sciences, University of Macau, Taipa, Macao; crystal199406@hotmail.com (J.W.); amywu1989@hotmail.com (M.-Y.W.); HuanxingSu@um.edu.mo (H.S.); JinjianLu@um.edu.mo (J.L.); XPChen@um.edu.mo (X.C.); 2 Center for Medical Genetics, School of Life Sciences, Central South University, Changsha 410008, Hunan, China; tanjieqiong@sklmg.edu.cn

**Keywords:** iNOS, NO, p62/SQSTM1, autophagy, macrophage

## Abstract

Nitric oxide (NO) is an important mediator of inflammation response and the production of NO has been linked to a variety of diseases, including tumors, inflammation and central nervous system diseases. In macrophages, a high level of NO is generated by iNOS during inflammatory responses triggered by cytokines or pathogens. Autophagy, a cellular bulk degradation process via lysosome, has been implicated in many disease conditions including inflammation. In this study, we have reported the previously unknown role of autophagy in regulating iNOS levels in macrophages, both under basal and Lipopolysaccharides (LPS)-induced conditions. Our data showed that iNOS levels accumulated upon autophagy inhibition and decreased upon autophagy induction. iNOS interacted and co-localized with autophagy receptor p62/SQSTM1, especially under LPS-stimulated condition in macrophages. Moreover, the immunostaining data revealed that iNOS also co-localizes with the autophagosome marker LC3 and lysosome marker LAMP1, especially under lysosomal inhibition conditions, indicating iNOS is an autophagy substrate. Finally, we showed that autophagy negatively regulated the generation of NO in macrophages, which is consistent with the changes of iNOS levels. Collectively, our study revealed a previously unknown mechanism by which autophagy regulates iNOS levels to modulate NO production during inflammation.

## 1. Introduction

NO is an important inflammation signaling molecule which is synthesized from L-arginine by nitric oxide synthase (NOS). The NOS enzymes family contains three isoforms: endothelial nitric oxide synthase (eNOS) mainly located at epithelial cells, neuronal nitric oxide synthase (nNOS) mostly distributed in neurons and inducible nitric oxide synthase (iNOS) widely expressed in immune cells, including macrophages, dendritic cells, microglia and T cells during inflammatory response [1]. iNOS level is extremely high in macrophages after cytokines or bacterial stimulating, generating large amount of NO in a short time to mediate inflammation response as well as cause cytotoxicity [2]. In addition to transcriptional and translational regulation, iNOS level can also be regulated via ubiquitin-proteasome system by degradation [3,4].

Autophagy is an intracellular process for lysosome degradation and it usually means “self-eating”. It originates from a bilayer membrane structure called the autophagosome, which forms an autolysosome after fusion with lysosomes for degradation [5]. Autophagy was initially considered to be non-selective, but recent studies have found that this process can be selective, partially via the autophagy receptor proteins [6], to precisely degrade target proteins [7]. In early studies, LPS has been found to induce autophagy in human macrophages and in the RAW 264.7 cell line via a Toll-like receptor 4 (TLR4)-dependent pathway [8,9]. P62 is the classic autophagy receptor which recruits cargos to the autophagosome for degradation. Inhibition of autophagy leads to intracellular p62 aggregation and the amount of p62 protein can be used as an indicator of autophagic flux [10]. P62 expression is increased after LPS stimulation and it plays important roles in inflammation-related pathways regulation [11,12].

Autophagy is involved in inflammation regulation by multiple mechanisms including: degradation of inflammasome, clearance of pathogens, cytokine secretion and antigen presentation [13]. In this study, we find a new mechanism by which autophagy regulates inflammation response by affecting the NO production via controlling autophagic degradation of iNOS. 

## 2. Materials and Methods

### 2.1. Reagents

Chloroquine (CQ), SAR405, Torin 1, Rapamycin, Lipopolysaccharides (LPS), 1400 w dihydrochloride and Cycloheximide were purchased from Sigma-Aldrich (St. Louis, MO, USA). Nitric oxide Griess reagent and RIPA buffer were obtained from Beyotime Biotechnology (Shanghai, China). Dynabeads protein Immunoprecipitation kit, TNF-α and IL-6 ELISA kit were from Thermo Fisher Scientific (Dreieich, Germany). The mouse monoclonal antibody against iNOS (Cat. No: 610328) was purchased from Biosciences (Heidelberg, Germany). The rabbit monoclonal anti-p62 for Western blot and immunofluorescence (Cat. No: ab109012) was purchased from Abcam (Cambridge, UK) and rabbit polyclonal anti-p62 for co-immunoprecipitation (Cat. No: P0068) was obtained from sigma. The rabbit polyclonal anti-LC3 (Cat. No: NB100-2220) for western blot was from Novus Biologicals (Littleton, CO, USA) and rabbit polyclonal anti-LC3 (Cat. No: PM036) for immunofluorescence staining was obtained from MBL International Corporation (Woburn, MA, USA). Anti-GAPDH (Cat. No: 5174) and anti-Lamp1 (Cat. No: 9091) were obtained from Cell Signaling Technology (Boston, MA, USA).

### 2.2. Cell Culture and Treatment

Raw 264.7 cells were cultured in incubator at 37 °C with 5% CO_2_ and 95% humidified atmosphere. The cells grew in Dulbecco’s Modified Eagle’s medium (DMEM, Gibco, Darmstadt, Germany), containing 10% fetal bovine serum (FBS, Gibco) and 1% penicillin/streptomycin. Bone-marrow-derived macrophages (BMDMs) were obtained from the femur and tibia of C57BL/6 mice. Bone marrow cells were obtained by flushing femur and tibia with 1 X HBSS. The flushed out-bone marrow cells were cultured for 7 days in Dulbecco’s Modified Eagle’s medium (DMEM), containing 10% fetal bovine serum (FBS), 1% penicillin/streptomycin and 10% L929 cell culture medium. After 7 days, above 97% cells that attached to the bottle of dish are macrophages and can be used for experiments. 

### 2.3. Western Blot Analysis

Raw 264.7 cells and BMDMs were seeded at a density of 4 × 10^5^ per well in 12-well plates overnight, then treated with autophagy inhibitors (Chloroquine, 30 µM; SAR405, 1 µM) and inducers (Torin 1,1 µM; Rapamycin, 1 µM) for 24 h. Under LPS stress condition, Raw 264.7 treated with LPS (200 ng/mL) for 12 h, then replaced with new medium and treated with autophagy inhibitors or inducers for another 12 h. Raw 264.7 cells and BMDMs were washed with ice-cold phosphate buffer saline (PBS) twice, then lysed with RIPA buffer (50 mM Tris-HCl pH 8.0, 0.1 M NaCl, 20 mM EDTA, 1% SDS, contained with protease and phosphatase inhibitor cocktails). The lysates were denatured at 99 °C in sample loading buffer, proteins (20 µg) were resolved with SDS-PAGE and then transferred to a polyvinylidene difluoride membrane. Membranes were blocked with 5% fat-free milk in Tris-buffed saline supplemented with 0.1% Tween-20 (TBST) for 1h at room temperature. After that, membranes were incubated with primary antibodies overnight at 4 °C. After washing with TBST for 30 min, membranes were incubated with HRP-conjugated secondary antibodies for 2 h at room temperature and washed for another 30 min. Finally, HRP substrate (GE healthcare) was used to detect blots through chemiluminescence. The densitometric analysis of western blotting bands were calculated via software (Image Lab 5.1, Bio-Rad, Munich, German). Briefly, the file of bands was import into the software, the bands to be quantified were selected in each group, and the densitometric value of each bands were subtracted with background value. The adjusted densitometric value of each band was firstly normalized with GAPDH to get the relative densitometric value, then all the relative densitometric values were normalized with that of CTRL group to obtain the fold of control value.

### 2.4. Immunofluorescence Assay

Raw 264.7 cells were plated on glass coverslips in 24-well plates. Under basal condition, cells were treated with CQ and SAR for 12 h. While under stress condition, cells were treated with LPS (1 µg/mL) for 12 h, then replaced with fresh medium and treated with autophagy inhibitors or inducers for another 12 h. After treatment, cells were fixed with 4% paraformaldehyde for 10 min, and permeabilized with 0.3% Triton X-100 for 15 min (Sigma-Aldrich). Cells then were blocked with 5% BSA (Beyotime Biotechnolog) for 2 h and stained with iNOS antibody (1:100, Santa cruz) or p62 (1:100, Abcam) antibody overnight at 4 °C. The iNOS and p62 signals were visualized by incubating with Alexa Fluor488 (green) and Alexa Fluor555 (red) conjugated secondary antibodies (1:500) for 2 h at room temperature. The nuclei were stained with Hoechst, and the coverslips were mounted with a fluoromount aqueous mounting medium (Sigma-Aldrich). Cells were visualized through a confocal fluorescence imagine microscope (Leica TCS SP8; Leica Microsystems, Bensheim, Germany). Hoechst, Fluor488 (Green) and Fluor555 (Red) fluorescence is excited under 405 nm, 488 nm, and 552 nm laser excitation, respectively, and used sequential scanning for image capture.

Colocalization is analyzed via Image J (National Institutes of Health, Bethesda, MD, USA), and image distribution in the article is refer to the previous study [14].

### 2.5. Co-Immunoprecipitation Assay

Under basal condition, Raw 264.7 cells were plated in 6-well plates overnight. Under stress condition, Raw 264.7 cells treated with LPS (1 µg/mL) for 12 h. Then cells were washed with ice-cold PBS and lysed with IP lysis buffer (10 mM Tris-HCl, pH 7.5, 2 mM EDTA, 1% NP40, 150 mM NaCl, supplemented with protease and phosphates inhibitor cocktail). The lysates incubated with anti-iNOS antibody (1:100, Cell Signaling Technology) or p62 antibody (1:100, Sigma) overnight at 4 °C. 25 µL protein-G conjugated magnetic beads (Dynabeads Protein Immunoprecipitation kit, Thermo scientific) was added into each IP tube and incubate at 4 °C for 6 h. The supernatants were removed by aspiration and the beads were washed three times with the lysis buffer. Finally, the beads were denatured at 99 °C in 1×sample loading buffer and the IP products were detected by western blotting as mentioned above. 

### 2.6. ELISA Assay

Raw 264.7 cells were seeded in 12-well plates. Treatment with LPS (200 ng/mL) for 12 h, then replaced with fresh medium and treated with autophagy inhibitors or inducers for another 12 h. 200 µL of medium supernatant was collected after treatment. The levels of TNF-α and IL-6 were determined with enzyme-linked immunosorbent assay (ELISA) kit (Thermo Fisher Scientific) according to the technical guide protocol. Samples were measured in a 96-well plate by a fluorometer equipped with 450 nm. The absorbance of the samples was valued by comparing with a simultaneously generated standard curve.

### 2.7. Nitric Oxide Detection Assay

Raw 264.7 cells were seeded in 12-well plates and treated with LPS (200 ng/mL) for 12 h, then the culture medium was replaced with fresh medium and treated with autophagy inhibitors, autophagy inducers or 1400 w (100 µM) for another 12 h. After treatment, the medium supernatant was collected to detect the production of nitric oxide by Griess reagent (Beyotime Biotechnology) according to the manufacturer’s protocols. Samples were plated in a 96-well plate by a fluorometer equipped with 540 nm emission filter. The nitrite concentration was calculated from a nitrite standard curve in this method.

### 2.8. Statistics

For comparing different groups, statistical analysis was performed by one-way ANOVA with Turkeys as post hoc tests. Data were analyzed with GraphPad Prism and statistically significant at *p* < 0.05.

## 3. Results

### 3.1. Autophagy Regulates the Level of iNOS in Macrophages

**Figure 1:**
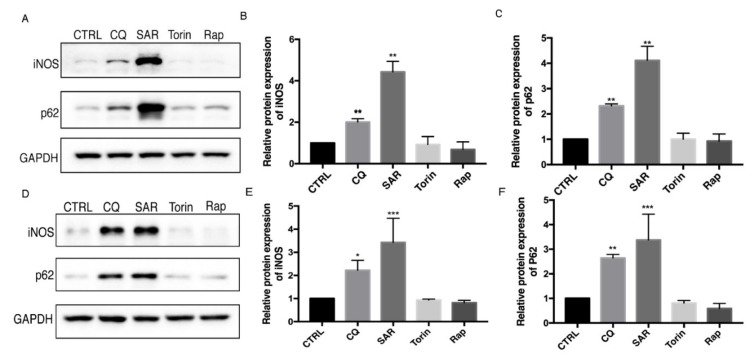
Autophagy inhibition lead to iNOS and p62 accumulation in Raw 264.7 and bone marrow-derived macrophages (BMDM). (**A**,**D**) Raw 264.7 cells (**A**–**C**) and BMDMs (**E**–**F**) were incubated with CQ (30 µM), SAR (1 µM), Torin (1 µM), Rap (1 µM) for 24h. iNOS, p62 levels were detected by western blotting. CTRL is blank control group with DMSO treatment. (**B**,**E**) Western blot analysis of iNOS in Raw 264.7 cells and BMDM cells under basal condition. (**C**,**F**) Western blot analysis of p62 in Raw 264.7 cells and BMDM cells under basal condition. (**B**–**F**) Values were analyzed from three individual experiments by GraphPad Prism, each experiment conducted three times. * *p* < 0.05, ** *p* < 0.01, *** *p* < 0.001. Error bars (mean ± SEM). One-way ANOVA with Turkeys as post hoc tests.

**Figure 2:**
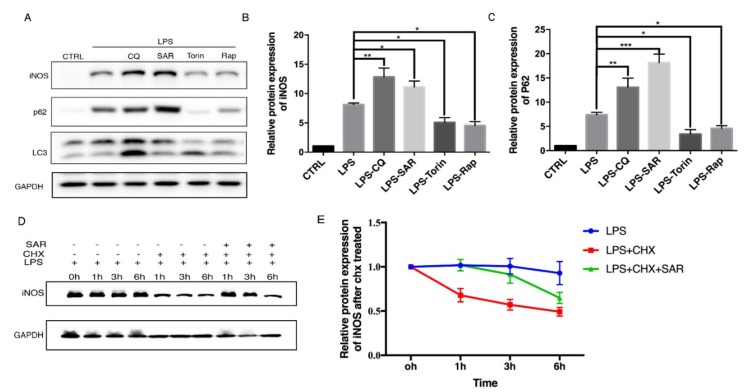
iNOS level is regulated by autophagy during LPS-stimulated condition. (**A**) Raw 264.7 cells were treated with LPS (200 ng/mL) for 12 h first, followed by removing the LPS and added CQ (30 µM), SAR (1 µM), Torin (1 µM), Rap (1 µM) for another 12 h. CTRL is blank control group with DMSO treatment. (**B**) Western blot analysis of iNOS in Raw 264.7 cells under LPS treatment. (**C**) Western blot analysis of p62 in Raw 264.7 cells under LPS treatment. (**D**) Raw 264.7 cells were treated LPS (200 ng/mL) for 12 h, then removed LPS added CHX (1 µg/mL) and autophagy related drugs for another 12 h. (**E**) Level of iNOS in Raw 264.7 cell after CHX treatment. (**B**–**E**) Values were analyzed from three individual experiments by GraphPad Prism, each experiment conducted three times. * *p* < 0.05, ** *p* < 0.01, *** *p* < 0.001. Error bars (mean ± SEM). One-way ANOVA with Turkeys as post hoc tests.

To explore the role of autophagy in iNOS degradation, Raw 264.7 macrophage cells were treated with autophagy inducers and inhibitors. Chloroquine (CQ) is an commonly used autophagy inhibitor by raising lysosome pH and decreasing autophagosome-lysosome fusion [15,16] and SAR405 is another autophagy inhibitor through PI3KC3 inhibition [17]. Torin 1 and rapamycin were commonly used as inducers of autophagy by inhibiting the mTOR pathway [18]. We found that the protein levels of iNOS are consistent with p62 level which dramatically accumulated upon autophagy inhibition in Raw 264.7 cells (Figure 1A–C). Consistently, we also detected the iNOS and p62 protein levels in bone marrow-derived macrophages (BMDMs) (Figure 1 D–F). We further sought to figure out whether the iNOS would display similar pattern after autophagy modulators treatment under the inflammation condition induced by LPS, a stronger inducer of inflammation and iNOS expression. After stimulation with LPS, the iNOS and p62 levels were dramatically increased. Interestingly, autophagy inhibitors further increased the iNOS and p62 levels while autophagy inducers decreased the iNOS and p62 levels in Raw 264.7 cells (Figure 2A–C). These data drove us to propose that iNOS is an autophagy substrate during inflammation. To understand whether the iNOS is degraded by autophagy, we used a eukaryote protein synthesis inhibitor cycloheximide to block protein translation and observed that the degradation rate of iNOS was impaired by addition of autophagy inhibitor SAR405 (Figure 2D,E). Thus, we found that iNOS can be degraded through the autophagy pathway, in a similar trend like p62 in Raw 264.7 cells.

### 3.2. iNOS Interacts and Colocalizes with Autophagy Receptor p62/SQSTM1 

**Figure 3:**
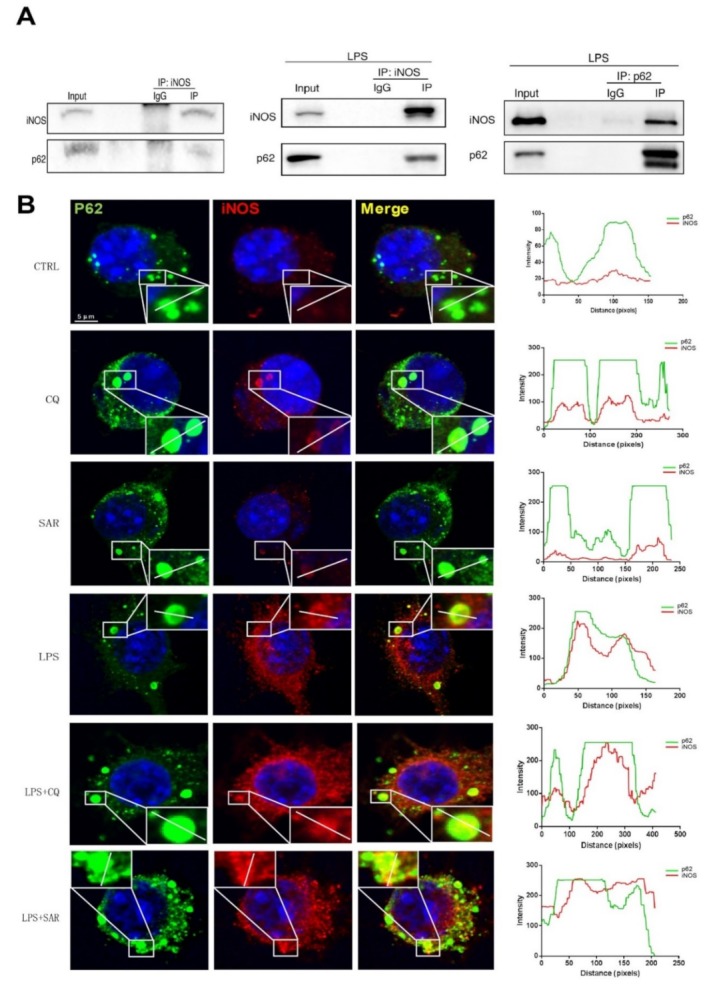
iNOS interacts and co-localizes with p62. (**A**) Raw 264.7 cells were treated with non-vehicle or LPS for 12 h. The co-immunoprecipitation analysis of protein extracts from Raw 264.7 expressing iNOS, p62. (**B**) Raw 264.7 cells were treated with CQ (30 µM) and SAR (1 µM) for 12 h. CTRL group is blank control group with DMSO treatment. The confocal analysis showed the interaction between p62 and iNOS under CQ and SAR treatment. Raw 264.7 cells were treated with LPS (1 µg/mL) 12 h, followed by removing LPS and add CQ (30 µM), SAR (1 µM) for another 12 h. the confocal images of Raw 264.7 cells which were stained with anti-iNOS and anti-p62 were captured by Leica microsystems. The results of co-localization were analyzed by Leica Application Suite X software and GraphPad Prism.

The observation that iNOS displayed similar trend as p62 under basal and inflammation conditions drove us to explore the potential relationship between iNOS and p62. We first examined whether iNOS interacts with p62 by immunoprecipitation. The result showed that iNOS antibody could pull down p62 in Raw 264.7 cells. To make the iNOS enriched obviously, we treated Raw 264.7 cells with LPS and the amount of p62 being pulled down dramatically increased under LPS-treated conditions. Meanwhile, p62 antibody could also pull down iNOS in Raw 264.7 cells after LPS stimulation (Figure 3A). To further support the interaction between iNOS and p62, the immunofluorescence assay was performed to observe the intracellular co-localization of p62 and iNOS under different conditions. Under basal condition, despite the low level of iNOS protein, we could see iNOS forms some puncta-like structure which partially co-localized with p62 in Raw 264.7 cells (Figure 3B). Since LPS can induce autophagy and inflammatory response-induced iNOS expression, the fluorescent signals of p62 and iNOS were both dramatically increased after LPS treatment. Compared to the LPS-only treatment, cells were treated with LPS plus CQ or SAR405 displayed stronger iNOS expression, which obviously colocalized with p62 puncta (Figure 3B). This result is consistent with the WB data. The co-localization efficiency was further demonstrated by line profiles. The data indicates that iNOS interacts with autophagy receptor p62 and forms puncta-like structure with p62 in macrophages.

### 3.3. iNOS Partially Colocalizes with Autophagosome Marker LC3 and Lysosome Marker LAMP1 

**Figure 4:**
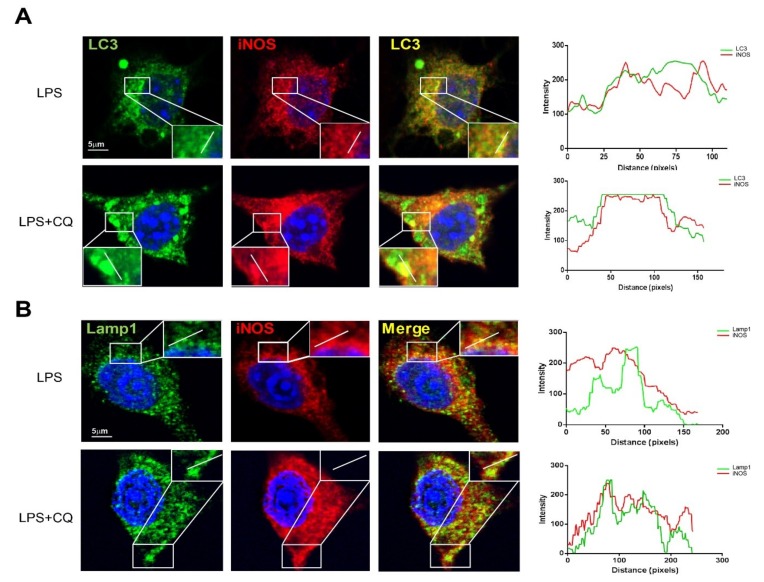
iNOS co-localizes with autophagosome and lysosome. (**A**) Raw 264.7 cells were treated with LPS (1 µg/mL) 12 h, followed by removing LPS and add CQ (30 µM) for another 12 h. CTRL group is blank control group with DMSO treatment. The confocal images of cells which were stained with anti-iNOS and anti-LC3. CTRL group is blank control group with DMSO treatment (**B**) Raw 264.7 cells were treated with LPS (1 µg/mL) 12 h, followed by removing LPS and add CQ (30 µM) for another 12 h. CTRL group is blank control group with DMSO treatment. The confocal images of cells which stained with anti-iNOS and anti-LAMP1 were captured by Leica microsystem. The results of co-localization were analyzed by Leica Application Suite X software and GraphPad Prism.

To further explore the relationship between iNOS with autophagy, we co-stained the iNOS with autophagosome marker LC3 and lysosome marker LAMP1, under LPS-only and LPS plus CQ-treated conditions. Interestingly, the co-localization between iNOS and LC3 or LAMP1 were obviously further increased after LPS plus CQ treatment compared to the LPS-only treatment in Raw 264.7 cells. The co-localization efficiency was further demonstrated by line profile (Figure 4A,B). The data reveals that iNOS can partially co-localize with autophagosome and lysosome under lysosome blockage condition. 

### 3.4. Modulation of Autophagy Affects NO Production and Inflammatory Cytokines Release

**Figure 5:**
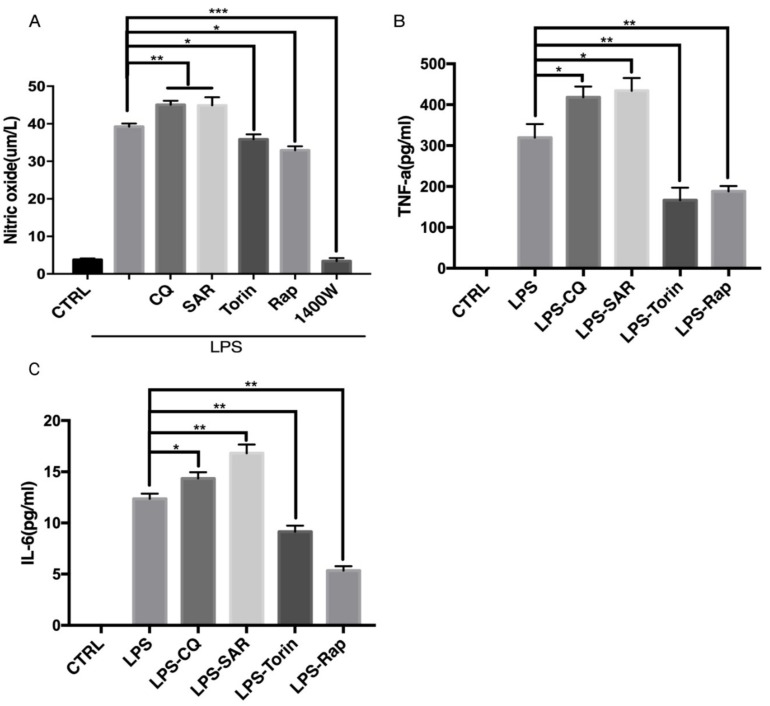
Regulation of autophagy affects NO production and inflammatory cytokines release. Raw 264.7 cells were treated with LPS (200 ng/mL) 12 h, followed by removing LPS and add CQ (30 µM), SAR (1 µM), Torin (1 µM), Rap (1 µM) 1400 w (100 µM) for another 12 h. (**A**) Detection of nitric oxide level from Raw 264.7 cells by Griess assay. (**B**) Detection of TNF-α level by ELISA assay (**C**) Detection of IL-6 level by ELISA assay. (**A**–**C**) CTRL group is blank control group with DMSO treatment. The results were analyzed from three individual experiments by GraphPad Prism, each experiment conducted three times. * *p* < 0.05, ** *p* < 0.01, *** *p* < 0.001. Error bars (mean ± SEM). One-way ANOVA with post hoc tests.

Level of iNOS determines the NO generation in macrophages. In the previous experiments, we showed that iNOS can be degraded by autophagy. We further sought to confirm if autophagy indeed regulates NO production in macrophages by regulating the iNOS degradation. The NO level is very low under basal condition, so we treated Raw 264.7 cells with LPS to induce inflammatory response for 12 h to induce the production of NO. Then we removed LPS-containing medium and added autophagy regulators for another 12 h. The NO level in the medium was measured by Griess assay [19]. As expected, the production of NO was decreased by the autophagy inducer Torin 1 and Rapamycin in Raw 264.7 cells (Figure 5A). Conversely, autophagy inhibitors CQ and SAR405 caused an increase in NO generation. iNOS inhibitor 1400w was used as a positive control to indicate that the production of NO after LPS treatment is completely from iNOS (Figure 5A). Besides that, we detected the inflammatory cytokines TNF-α and IL-6 in medium after LPS treatment by ELISA assay (Figure 5B,C). Interestingly, autophagy inhibitors CQ and SAR405 could increase the cytokines level while autophagy inducers Torin 1 and Rapamycin inhibited cytokines release, in a similar trend as iNOS and NO. These findings further revealed that autophagy could regulate the degradation of iNOS and production of NO, as well as the release of pro-inflammatory cytokines. 

## 4. Discussion

Autophagy is a recycling mechanism in eukaryotes and plays a key role in embryonic development, cell defending and survival. Under stress, the cells initiate the autophagy to remove damaged proteins [20], organelles or invading pathogens [21], through the lysosomal pathway and recycle the degradation products to cope with the adverse environments [22]. Inflammation is a defending response normally activated by microbial pathogen infection or tissue damage [23], which will recruit immune cells such as neutrophils, macrophages and monocytes to the lesion site [24]. Defects of autophagy pathway are closely related to the development of inflammatory diseases including Crohn’s disease [25], rheumatoid arthritis and cancer [26]. In this study, we firstly found that upon autophagy induction or inhibition, iNOS levels displayed similar change pattern as autophagy substrate p62, indicating iNOS is possible to be the substrate of autophagy. Secondly, further experiments of immunoprecipitation and Immunofluorescence assays confirmed the interaction and colocalization between iNOS and p62, especially under LPS-simulated condition. iNOS also partially colocalizes with autophagosome marker LC3 and lysosome marker LAMP1, implicating that iNOS can be degraded via autophagy. At last, nitric oxide, the product of iNOS, displayed same trend as iNOS during autophagy induction and inhibition. The results reveal that iNOS regulation via autophagy can be an aspect to explain the autophagy function in inflammation. All the above data demonstrate that iNOS interacted with autophagy receptor p62 and degraded by autophagy in macrophages.Our studies revealed that iNOS can be degraded by autophagy in macrophages and inhibition of autophagy resulted in accumulation of iNOS and its product NO. The results also reveal a novel mechanism by which autophagy regulates inflammation. 

Autophagy is deeply implicated in inflammatory response in macrophages with diverse mechanisms, such as degradation of inflammasome, clearance of pathogens, cytokine secretion and regulation of NF-kappa B signaling [13]. Although there are discoveries revealing that activation of autophagy pathway would affect level of iNOS and inflammatory-related cytokines production in microglia cells [27], a clear elaboration of the relationship between autophagy and iNOS still lack [28]. Our results revealed that iNOS interacts with autophagy receptor p62 and is degraded by autophagy in macrophages, suggesting that autophagy could regulate the iNOS protein level, as well as the NO production during inflammation. This mechanism could partially explain why autophagy regulation has been linked to iNOS level and NO production level change in a previous study [29]. However, our study does not exclude the potential role of autophagy in the transcriptional regulation of iNOS level by other signaling pathways including NF-kappa B. Cycloheximide (CHX) is known to suppress protein synthesis and has been widely used to analyze the protein degradation rate. We can see the rapid degradation of iNOS after CHX treatment, while inhibition of autophagy by SAR405 partially blocked iNOS degradation, indicating that iNOS can be degraded via autophagy. Considering that CHX can inhibit autophagy in the early stage via inhibition of newly synthesized protein needed in autophagy or activation of mTORC1 activity [30], the inhibition effect of SAR405 on iNOS degradation may be more obvious than it appeared because CHX may already partially impair the autophagic degradation of iNOS. As one necessary signaling molecules, NO plays an important role in physiology and pathology [2]. The overexpression of iNOS and NO have been shown to result in various damage in inflammation microenvironment [31]. Our study thus offers a new strategy to control iNOS level and NO production during inflammatory process via regulating autophagy. Interaction between iNOS and autophagy receptor p62 reveals a selective degradation of iNOS via autophagy. However, there is no evidence to support the direct interaction between iNOS and p62, further study will be performed to understand whether iNOS directly interacts with p62 and the interaction domains, as well as to understand whether the interaction can be regulated via post-translational modifications. Furthermore, whether iNOS-p62 interaction will affect the function of p62 worth further examination.

iNOS level keeps at a relative low level and can be strongly up-regulated during inflammation. However, prolonged iNOS upregulation will generate high-level NO which may cause tissues damage, thus efficient degradation of iNOS is a critic mechanism for elimination of inflammation [32]. iNOS has been revealed to be degraded by UPS [33,34] and our data revealed for the first time that iNOS can also be degraded by autophagy, in a selective manner via the interaction with p62. Compared with the dramatic accumulation of iNOS when proteasome is inhibited [35], the increase of iNOS level is moderate during autophagy inhibition. Furthermore, under protein translation inhibition condition, the autophagy inhibition can only partially impair the iNOS degradation. These data suggest that UPS may play a major role for iNOS degradation while autophagy can be a fine-turn for selective iNOS degradation. The collaboration of UPS and autophagy pathway may help eliminate the NO over-production during inflammation via promoting the iNOS degradation.

## Abbreviations

iNOS, inducible nitric synthase; eNOS, endothelial nitric synthase; nNOS, neuronal nitric synthase; NO, nitric oxide; CQ, Chloroquine; LPS, Lipopolysaccharides; BMDM, bone marrow-derived macrophages.
